# Acute Hypoxemic Respiratory Failure in Children at the Start of COVID-19 Outbreak: A Nationwide Experience

**DOI:** 10.3390/jcm10194301

**Published:** 2021-09-22

**Authors:** Yolanda M. López-Fernández, Amelia Martínez-de-Azagra, José M. González-Gómez, César Pérez-Caballero Macarrón, María García-González, Julio Parrilla-Parrilla, María Miñambres-Rodríguez, Paula Madurga-Revilla, Ana Gómez-Zamora, Patricia Rodríguez-Campoy, Juan Mayordomo-Colunga, Laura Butragueño-Laiseca, Rocío Núñez-Borrero, Jesús M. González-Martín, Arthur S. Slutsky, Jesús Villar, on behalf the Prevalence and Outcome of Acute Hypoxemic Respiratory Failure in Children (PANDORA-CHILD) Network

**Affiliations:** 1 Pediatric Intensive Care Unit, Hospital Universitario de Cruces, Biocruces-Bizkaia Health Research Institute, 48903 Barakaldo, Spain; 2 Pediatric Intensive Care Unit, Hospital Universitario Niño Jesús, 28009 Madrid, Spain; ameliamartinezdeazagra@gmail.com; 3 Pediatric Intensive Care Unit, Hospital Regional Universitario de Málaga, 29010 Málaga, Spain; josemagogo@hotmail.com; 4 Pediatric Intensive Care Unit, Hospital Universitario Ramón y Cajal, 28034 Madrid, Spain; cesarperezcaballero@yahoo.es; 5 Pediatric Intensive Care Unit, Complejo Hospitalario de Burgos, 09006 Burgos, Spain; lapuris@hotmail.com; 6 Pediatric Intensive Care Unit, Hospital Universitario Virgen del Rocío, 41013 Sevilla, Spain; julioparill@hotmail.com; 7 Pediatric Intensive Care Unit, Hospital Universitario Virgen de la Arrixaca, 30120 Murcia, Spain; mariamiro@gmail.com; 8 Pediatric Intensive Care Unit, Hospital Universitario Miguel Servet, 50009 Zaragoza, Spain; paumare@hotmail.com; 9 Pediatric Intensive Care Unit, Hospital Universitario La Paz, 28046 Madrid, Spain; agzamora77@gmail.com; 10 Pediatric Intensive Care Unit, Hospital Universitario Puerta del Mar, 11009 Cádiz, Spain; p.r.campoy@gmail.com; 11 Pediatric Intensive Care Unit, Hospital Universitario Central de Asturias, Instituto de Investigación del Principado de Asturias, 33006 Oviedo, Spain; jmcolunga@hotmail.com; 12 CIBER de Enfermedades Respiratorias, Instituto de Salud Carlos III, 20029 Madrid, Spain; jesus.villar54@gmail.com; 13 Pediatric Intensive Care Unit, Hospital Universitario Gregorio Marañón, 28009 Madrid, Spain; laura_bl@hotmail.com; 14 Pediatric Intensive Care Unit, Hospital Universitario Materno-Infantil, 35016 Las Palmas de Gran Canaria, Spain; rocio.nunez.borrero@gmail.com; 15 Research Unit, Hospital Universitario Dr. Negrín, 35019 Las Palmas de Gran Canaria, Spain; josu.estadistica@gmail.com; 16 Interdepartmental Division of Critical Care Medicine, University of Toronto, Toronto, ON M5S 1A4, Canada; arthur.slutsky@unityhealth.to; 17 Keenan Research Center for Biomedical Sciences, Li Ka Shing Knowledge Institute, St. Michael’s Hospital, Toronto, ON M5B 1W8, Canada; 18 Multidisciplinary Organ Dysfunction Evaluation Research Network (MODERN), Research Unit, Hospital Universitario Dr. Negrín, 35019 Las Palmas de Gran Canaria, Spain

**Keywords:** children, acute hypoxemic respiratory failure, acute respiratory distress syndrome, coronavirus disease 2019, mechanical ventilation

## Abstract

Study design: This is a prospective, multicenter, and observational study with the aim of describing physiological characteristics, respiratory management, and outcomes of children with acute hypoxemic respiratory failure (AHRF) from different etiologies receiving invasive mechanical ventilation (IMV) compared with those affected by SARS-CoV-2. Methods and Main Results: Twenty-eight patients met the inclusion criteria: 9 patients with coronavirus disease 2019 (COVID-19) and 19 patients without COVID-19. Non-COVID-19 patients had more pre-existing comorbidities (78.9% vs. 44.4%) than COVID-19 patients. At AHRF onset, non-COVID-19 patients had worse oxygenation (PaO_2_/FiO_2_ = 95 mmHg (65.5–133) vs. 150 mmHg (105–220), *p* = 0.04), oxygenation index = 15.9 (11–28.4) vs. 9.3 (6.7–10.6), *p* = 0.01), and higher PaCO_2_ (48 mmHg (46.5–63) vs. 41 mmHg (40–45), *p* = 0.07, that remained higher at 48 h: 54 mmHg (43–58.7) vs. 41 (38.5–45.5), *p* = 0.03). In 12 patients (5 COVID-19 and 7 non-COVID-19), AHRF evolved to pediatric acute respiratory distress syndrome (PARDS). All non-COVID-19 patients had severe PARDS, while 3 out of 5 patients in the COVID-19 group had mild or moderate PARDS. Overall Pediatric Intensive Care Medicine (PICU) mortality was 14.3%. Conclusions: Children with AHRF due to SARS-CoV2 infection had fewer comorbidities and better oxygenation than patients with non-COVID-19 AHRF. In this study, progression to severe PARDS was rarely observed in children with COVID-19.

## 1. Introduction

The novel coronavirus disease 2019 (COVID-19) has affected the population around the globe. The first pneumonia cases were reported in Wuhan (central China) in December 2019 [1,2], but the infection spread extremely rapidly and the COVID-19 pandemic was declared in March 2020 [3]. Early reports from China focused on adults because initially, children experienced disease with milder symptoms [4,5]. However, since late April 2020, a multisystem inflammatory syndrome associated with COVID-19 (MIS-C) [6,7,8,9,10,11,12] in children was reported from a number of countries. Most children with this syndrome had fever, gastrointestinal, mucocutaneus, hematological and respiratory symptoms, and cardiovascular involvement appeared to be responsible for disease severity.

Adults with COVID-19 developed acute hypoxemic respiratory failure (AHRF) with an associated high mortality requiring intubation and invasive mechanical ventilation (IMV) [13,14,15]. However, in children with respiratory failure requiring IMV due to COVID-19, overall mortality was considerably lower [4,5,8,9,10,11,12]. Unlike adults, there is a paucity of studies comparing children with AHRF associated with COVID-19 versus patients with AHRF due to other causes.

The present study includes an analysis of children admitted with AHRF and receiving IMV in a Spanish network of Pediatric Intensive Care Units (PICUs) during the first wave (March/April 2020) of the COVID-19 outbreak. As one of the three hardest hit countries in the world with an overwhelming pressure on its health care system, Spain has a population of 47,351,567 inhabitants [16], and officially recorded 224,510 confirmed cases of SARS-CoV-2 infections and 25,900 deaths during this two-month period [17]. The primary objective of this study is to describe the physiologic characteristics and ventilatory management of children with AHRF caused by COVID-19 in the Spanish pediatric population. Secondary objectives were: (1) to compare the characteristics of AHRF in patients with COVID-19 with a concurrent cohort of children who developed AHRF from other etiologies, and (2) to determine and compare the outcomes observed in both cohorts.

## 2. Material and Methods

This study was approved by the Ethics Committees at Cruces Hospital (PI201935) and Niño Jesús Hospital (R-0026/19), the coordinating centers, and was adopted by all participating centers as required by Spanish regulations. This study followed the “Strengthening the Reporting of Observational Studies in Epidemiology (STROBE)” guidelines for observational cohort studies [18] Electronic Supplementary Material (ESM).

### 2.1. Study Design and Patients

This study includes a specific analysis of children admitted with AHRF and receiving IMV in a network of Spanish PICUs during the first wave (March/April 2020) of the SARS-CoV-2 pandemic. The present study represents a partial analysis of the ongoing PANDORA-CHILD (NCT04791501), a prospective, multicenter, observational study focused on prevalence and outcomes of AHRF in children in Spain over 1 year (see ESM for details). From a total of 40 PICUs in Spain, 22 PICUs agreed to participate. However, during the study period, 6 of those PICUs served as adult ICUs due to the COVID-19 pandemic. Therefore, eligible patients came from eight geographical catchment areas of Spain covered by a network of 16 hospitals (162 pediatric critical care beds; Appendix A). The total catchment population for these 16 hospitals is 21,465,569, with 3,383,356 under the age of 16 [16]. All consecutive patients from 7 days to 16 years of age admitted to the PICU during a 2-month period (1 March–30 April 2020) were enrolled into the study if they fulfilled all the following inclusion criteria: (i) acute episode of respiratory failure (within 7 days of the clinical insult or worsening clinical status), (ii) invasively ventilated, (iii) PaO_2_/FiO_2_ ≤ 300 mmHg (or SpO_2_/FiO_2_ ≤ 264 if PaO_2_ was unavailable and provided that SpO_2_ ≤ 97%) [19] (iv) positive end-expiratory pressure (PEEP) ≥ 5 cmH_2_O and FiO_2_ ≥ 0.3. Patients were included even if cyanotic congenital cardiac disease, cardiac failure, left ventricular hypertension, or intravascular volume overload were the cause of AHRF. Patients solely receiving non-invasive respiratory support were excluded. This study was considered an audit, and informed consent was waived, although three local sites required written parental consent as per the institutions’ policies (ESM).

### 2.2. Data Collection

All PICU admissions were screened daily for AHRF. Onset of AHRF was defined as the day on which the patient first met all inclusion criteria. A transient fall in oxygenation resulting from an acute event unrelated to the disease process (such as obstruction of the endotracheal tube by secretions or after suctioning or after an inadvertent disconnection of the ventilator, patient-ventilator asynchronies, sudden pneumothorax, and hemodynamic instability) was not considered as a qualifier for AHRF. All data were collected on standardized forms. Demographics, comorbidities, reason for initiation of IMV, arterial blood gases, laboratory, radiographic, hemodynamic, and ventilator data were collected at study entry and during the first three days of AHRF diagnosis (T0 or time of inclusion into the study, 24 h, days 2 and 3; ESM 2). Tidal volume (VT) was calculated on the basis of predicted body weight (PBW) [20]. Plateau pressure (Pplat) was determined after the application of a 0.5- to 1.0-sec end-inspiratory hold in absence of spontaneous breaths. Static respiratory compliance (Crs) was calculated from registered variables and reported in mL/cmH_2_O/kg. Driving pressure was calculated as the difference between Pplat and PEEP [21]. Patients were classified into two groups: (i) confirmed or highly suspected infection for SARS-CoV-2 (COVID-19 patients), (ii) any other etiology (non-COVID-19 patients). Patients meeting Pediatric Acute Respiratory Sistress Syndrome (PARDS) criteria were stratified into mild, moderate, and severe according to the Pediatric Acute Lung Injury Consensus Conference (PALICC) definition [22]. Microbiological diagnosis of SARS-CoV-2 infection (PCR on nasopharyngeal swab samples or serological tests) was recorded. Patients were classified as having multisystem inflammatory syndrome associated with COVID-19 (MIS-C) according to Royal College of Pediatrics and Child Health criteria [23]. Outcome data from each subgroup were analyzed and compared. Severity of illness was measured using the Pediatric Risk of Mortality (PRISM) III score [24] after PICU admission, and extrapulmonary organ failure included in the Pediatric Logistic Organ Dysfunction (PELOD) 2 score was documented daily [25].

Ventilator-free days (VFDs) were determined by subtracting total ventilator days from 28 days in survivors [26]. All patients with total ventilator days of ≥28 and all PICU non-survivors were assigned VFD = 0. All patients were followed until PICU and hospital discharge. PICU and hospital mortality were recorded.

Data were collected and stored at each center and sent to study coordinators at the time of patient’s hospital discharge. Age, gender, PBW, risk factors, oxygenation (PaO_2_/FiO_2_, SpO_2_/FiO_2_, oxygenation index (OI), oxygenation saturation index (OSI)), and pediatric severity scores were checked and reassessed at the coordinating centers. If inconsistencies were found, the site principal investigator was contacted to clarify corrections were made accordingly.

### 2.3. General Management

Although patient care was not strictly protocolized, physicians were asked to follow the current standards of pediatric critical care management (ESM). For ventilatory management, it was recommended that all patients be ventilated with a VT of 6–8 mL/kg PBW, at a ventilatory rate to maintain PaCO_2_ at 35–50 mmHg, a Pplat <30 cmH_2_O, and PEEP and FiO_2_ combinations to maintain PaO_2_ >60 mmHg or SpO_2_ >90%.

### 2.4. Statistical Analysis

We used descriptive statistics to summarize binary (number and percentage) and continuous (median and P_25_–P_75_) variables. The Shapiro-Wilk test was used to check the normality of the data. We compared variables across groups using a Mann-Whitney test for numerical variables and Fisher’s exact test for categorical variables. A multiple linear regression for paired data was used to test the evolution of different numerical variables as a function of time and group (COVID-19 vs. non-COVID-19 patients). We assessed the probability of survival at day 60 using the Kaplan-Meier method and analyzed with the log-rank test and univariable Cox regression analysis. Patients with the complementary outcome were right-censored at the longest recorded length of stay. All time to events were defined from day 1 of IMV. Since this was an observational study with no harm and no benefit, we aimed to recruit patients with no pre-defined sample size. Missing data were not imputed. We considered 2-sided *p*-values < 0.05 to indicate statistical significance. Analyses were performed using R software, version 4.0.2 (R Foundation for Statistical Computing, Vienna, Austria).

## 3. Results

**Figure 1:**
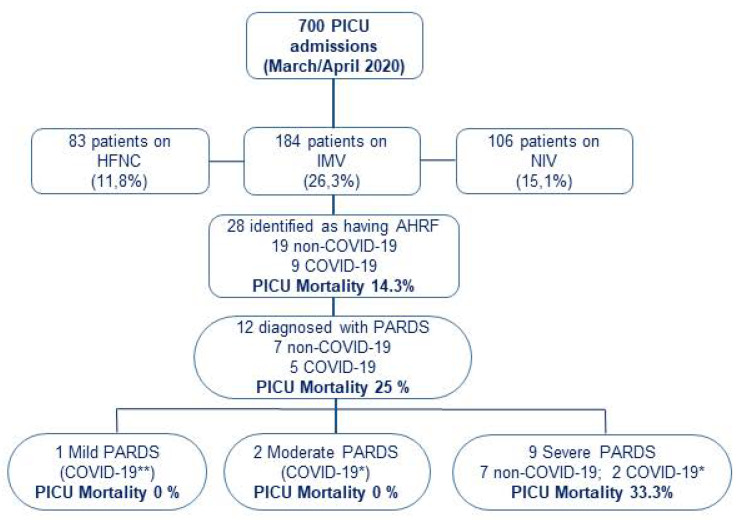
Patients flowchart. Patients who fulfilled study criteria of PaO_2_/FiO_2_ ≤ 300 mmHg or SpO_2_/FiO_2_ ≤ 264 mmHg on invasive mechanical ventilation with a positive end-expiratory pressure (PEEP) ≥ 5 cmH_2_O, and with a FiO_2_ ≥ 0.3 were included in the study. Patients who met PARDS criteria were stratified according to PALICC PARDS definition. All other patients were ineligible. * COVID-19: COVID-19 patients without MIS-C. ** COVID-19: COVID-19 patients with MIS-C. PICU: Pediatric Intensive Care Unit. HFNC: High Flow Nasal Cannula; IMV: Invasive Mechanical Ventilation; NIV: Non-invasive Mechanical Ventilation; AHRF: Acute Hypoxemic Respiratory Failure; PARDS: Pediatric Acute Respiratory Distress Syndrome.

During the study period, a total of 700 children were admitted to participating PICUs: 373 patients required respiratory support for >24 h (189 with non-invasive respiratory support -either high-flow nasal cannula or non-invasive ventilation-, and 184 required IMV; Figure 1). Only those on IMV were included in further analysis. A total of 28 patients met inclusion criteria for AHRF, resulting in a prevalence of 4% (95% CI: 2.5–5.5) of total PICU admissions and 15.2% (95% CI 10–20.4) among those on IMV. The etiology of AHRF was attributed to SARS-CoV-2 in nine patients (four patients also met MIS-C criteria) and to other causes in nineteen patients. Twelve patients met criteria for PARDS: five in the COVID-19 group and seven in the non-COVID-19 group. All PARDS in non-COVID-19 patients were stratified as severe at ARDS onset, compared with two COVID-19 patients.

### 3.1. Patient Characteristics

**Figure 2:**
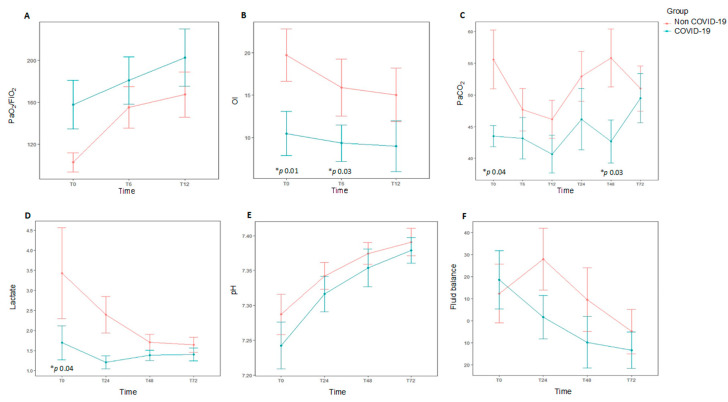
Data at different time points over the first 72 h for each study group comparing subjects diagnosed with acute hypoxemic respiratory failure (AHRF) and COVID-19 infection (*n* = 9) to those with ARHF without COVID-19 infection (*n* = 19). * *p* value representing statistical significance. Top: Oxygenation metrics over the 12 first hours after onset of AHRF, and daily ventilation metrics over the first 72 h of AHRF. (**A**). PaO_2_/FiO_2_ ratio. (**B**). Oxygenation index (OI). (**C**). PaCO_2_. Bottom: Daily hemodynamic/metabolic data over the first 72 h for subjects diagnosed with AHRF with COVID-19 infection. (**D**). Lactate (**E**). pH. (**F**). Fluid balance. Oygenation index: FiO_2_ × median airway pressure × 100/PaO_2_.

Median age was 26 months (P_25_–P_75_ 5–108) and 64.3% (18/28) were males. Although the median age was lower in non-COVID-19 patients (15 months vs. 106 months), the difference did not reach statistical significance (*p* = 0.17). Demographics and clinical characteristics at baseline are shown in Table 1. The diagnosis of respiratory failure was mainly performed using chest X-rays in most patients (24/28, 85.4%). Only two patients were diagnosed by CT scan. A total of 19 out of 28 patients (67.8%) had a pre-existing comorbidity. The most common causes of AHRF were pneumonia or lower respiratory tract infection (14/28, 50%), septic shock (4/28, 14.3%), and central nervous system disorders (4/28, 14.3%). In patients with PARDS, the main risk factor was pneumonia (9/12, 75%), whereas sepsis was uncommon (2/12, 16.6%). Non-COVID-19 AHRF patients had a tendency to have comorbidities (*p* = 0.09), the most common being chronic neurologic disease. Non-COVID-19 patients were more likely to have worse oxygenation at study entry (PaO_2_/FiO_2_ 95 mmHg (65.5–133) vs. 150 mmHg (105–220), *p* = 0.04), OI 15.9 (11–28.4) vs. 9.3 (6.7–10.6), *p* = 0.01) than COVID-19 patients. There was a higher PaCO_2_ in non-COVID-19 patients (56 mmHg (48–71) vs. 43.5 (40–46), *p* = 0.04] that remained higher at 48 h [56 mm Hg (43–59) vs. 43 (39.5–46), *p* = 0.03) (Table 2, Figure 2). In patients with PARDS (*n* = 12), non-COVID-19 patients had greater severity according to PALICC criteria (*p* = 0.04).

### 3.2. Ventilatory Settings and Adjunctive Measures

**Figure 3:**
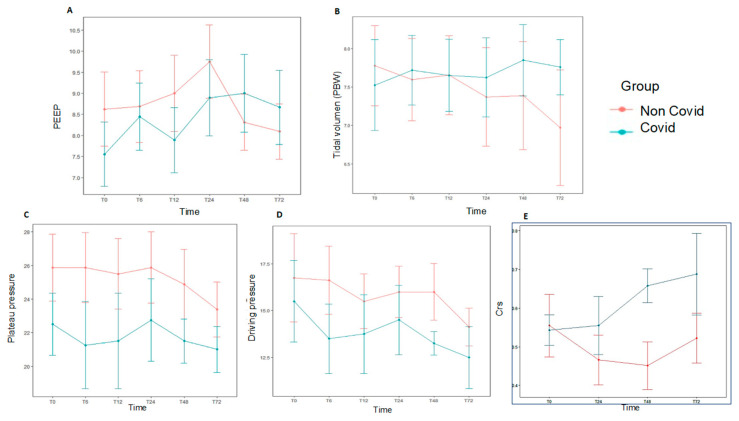
Daily ventilation data at different time points over the first 72 h for each study group, subjects diagnosed with AHRF and COVID-19 (*n* = 9) compared with those with AHRF without COVID-19 (*n* = 19). There was no statistical significance in the data shown. (**A**). PEEP. (**B**). Tidal volume accorfing to PBW. (**C**). Plateau pressure. (**D**). Driving pressure. (**E**). Static respiratory compliance (Crs). AHRF: Acute Hypoxemic Respiratory Failure. Crs: static respiratory compliance; PBW: predicted body weight.

At baseline, median VT was 7.4 mL/kg PBW (P_25_–P_75_ 6.1–9), median PEEP was 8 cmH_2_O (P_25_–P_75_ 6–9), and median Pplat and driving pressure were 25.5 (P_25_–P_75_ 21–29) cmH_2_O and 16 cmH_2_O (P_25_–P_75_ 13–20.5), respectively (Supplemental Table S1). Pplat and driving pressure were obtained in 20 patients, and no significant differences were found between COVID-19 and non-COVID-19 groups. Daily ventilation data over the first 72 h are shown in Figure 3 and Supplemental Table S2. There were no significant differences in both groups for hemodynamic data, fluid balance, need of blood products or replacement renal therapy (Supplemental Table S3, Figure 2). The most common modes of MV at the time of AHRF diagnosis were volume assist-control (18/28, 64.3%) and pressure-regulated volume control (9/28, 32.1%).

Prior to enrolment into the study, 39.2% patients (11/28) were managed with non-invasive ventilation and 28.6% (8/28) received high-flow nasal oxygen. Neuromuscular blockade (NMB) was used in two-thirds of patients within 24 h of enrolment, and two out of three patients received corticosteroids (Table 3). Other adjunctive therapies included nitric oxide (iNO; seven patients, 25%) prone positioning (PP; six patients, 21.4%) and recruitment maneuvers (RM; five patients, 17.8%). Five patients receiving iNO and five patients with PP had severe ARDS. All patients managed with recruitment maneuvers (RM) were also stratified as severe ARDS. Three patients (10.7%) diagnosed with severe PARDS were supported with ECMO. No differences were observed in non-COVID-19 patients compared with COVID-19 patients in terms of the use of adjunctive therapies.

### 3.3. Clinical Outcomes

**Figure 4:**
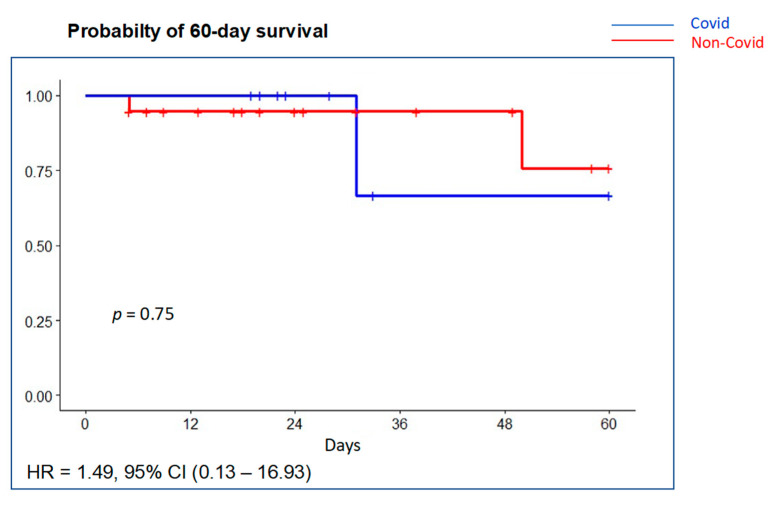
Probability of survival at 60 days using Kaplan–Meier curves with univariable Cox regression. There was no significant difference in survival between the COVID-19 group (blue line) compared with the non-COVID-19 group (red line).

Pneumothorax developed in two patients (10.7%), two of them with a VT >8 mL/kg PBW, without differences between groups. Mean ventilator-free days were 19 days (P_25_–P_75_ 5.5–24) with no significant differences between groups (Table 4). Mean duration of PICU and hospital stay was 16.5 (P_25_–P_75_ 12–26) and 23.5 (19–41) days, respectively. Overall PICU mortality was 14.3% (4/28) and in-hospital mortality was 17.9% (5/28). Probability of survival at 60 days is shown in Figure 4. Most deaths occurred in the ICU (4 out of 5, 80%). Although PICU deaths were two-fold greater in the COVID-19 group (22.2% (2 of 9) vs. 10.5% (2 of 19)), it did not reach statistical significance (*p* = 0.75). Multiple system organ failure was the cause of death in two patients (one COVID-19 patient) and refractory hypoxemia in two patients (one COVID-19 patient); brain death was declared in one patient.

## 4. Discussion

In this multicenter, observational study, we report a series of 28 children requiring IMV due to AHRF of any etiology at a time when the first wave of the COVID-19 pandemic devastated the Spanish Healthcare System. The major findings of this study are that pediatric AHRF patients with COVID-19 had fewer comorbidities and better oxygenation than patients with non-COVID-19 AHRF. Of note, all non-COVID-19 patients who progressed to ARDS developed severe PARDS, whereas most patients in the COVID-19 cohort had mild and moderate PARDS. We found no differences in ventilator management, use of ancillary therapies, or outcomes between groups. To our knowledge, no studies have compared children who require IMV due to COVID-19 with children with AHRF secondary to other causes.

During the study period, 13% of all hospitalized pediatric COVID-19 patients in Spain ended up in the PICU, as reported by the Spanish National Health System [27]. The present study can be considered close to being nationwide since only five additional mechanically ventilated children due to COVID-19 were admitted during the 2-month period into Spanish PICUs that were not sites in the present study [28]. Our study included intubated patients with COVID-19 who met predefined AHRF criteria, accounting for 1.3% of all patients admitted to participating PICUs and 32.1% of patients with AHRF and IMV. For the total number of COVID-19 children admitted in Spanish PICUs, our cohort represents 18% of the total and 64.3% of all children intubated [28]. These figures represent a more severe impact of COVID-19 in children than initially reported at the beginning of the pandemic. In a larger series, Dong et al. reported respiratory failure/ARDS in 0.6% of children with suspected or confirmed disease [5], without specifying what ratio corresponds to patients requiring PICU admission. In hospitalized children, Wang et al. published a series of 33 children, none of them requiring non-invasive or invasive respiratory support [29]. However, early reports from US and Canada showed that 38% of children admitted to PICUs for COVID-19 required IMV [30]. In United Kingdom, Swam et al. reported a prevalence of PICU admissions for SARS-CoV2 infection of 18%, similar to our series, although IMV was more often required (50%) [10]. Feldstein et al. reported 651 children admitted to PICUs, 25% of whom required IMV, and found a higher ratio of intubated patients in non-MIS-C COVID-19 compared with MIS-C patients (33% vs. 23.8%) [11].

Although not statistically significant (likely due to our small sample size), patients with AHRF due to a cause other than COVID-19 were younger and had more comorbidities than COVID-19 patients. However, three patients less than 1 year old in the COVID-19 cohort had severe respiratory compromise. This is consistent with several studies describing the seriousness of COVID-19 in some children. Severe respiratory impairment associated with SARS-CoV-2 infection has been described as more frequent in younger children [5,31] similar to our series, while MIS-C, more prevalent in older children, has been recognized in many PICU admissions for COVID-19 since April 2020 [9,10]. Pre-existing comorbidities, especially immunosuppression or chronic lung disease, are commonly seen in children admitted to PICUs with AHRF and/or PARDS [32,33]. We confirmed these findings in our cohort of non-COVID-19 patients, predominantly chronic neurological disease. In the COVID-19 cohort, the association with comorbidities was also present, although lower in the subpopulation of MIS-C patients [9,10]. Of note, among nine patients in the COVID-19 group, four developed MIS-C without having pre-existing diseases.

We found that patients with AHRF caused by non-COVID-19 diseases had worse oxygenation at AHRF onset. We observed confirmed more abnormal ventilatory data, which were maintained throughout the course of mechanical ventilation (as shown in Table 2). This may reflect that COVID-19 patients were promptly intubated mainly due to hemodynamic alterations and/or by the initial recommendation for early intubation to avoid a greater risk of virus dissemination associated with non-invasive respiratory support [34]. The prevalence of PARDS in our patient population is different than in previous studies [35]. This could be partially explained because we only included patients with IMV and that the strict lockdown isolation measures dramatically decreased the incidence of lower respiratory tract infections.

Contrary to adults, only an extremely small percentage of children infected by SARS-CoV-2 had severe respiratory symptoms and progression to ARDS [5,36]. There is paucity of data on the occurrence of PARDS in pediatric patients with COVID-19. As part of a large-scale multicenter study including patients <18 years from Europe, Asia, and US, Duarte-Salles et al. compared COVID-19 patients with a prior cohort of influenza patients and observed that while the prevalence of influenza-associated PARDS was 0.1%, the prevalence of PARDS among children with COVID-19 was 2.5%, suggesting a greater severity of respiratory involvement in COVID-19 [37]. Similarly, a European multicenter study involving 532 children with COVID-19 found that only 25 patients (4%) required IMV and 10 of them had PARDS [38]. However, this series does not differentiate whether these patients had predominantly respiratory involvement or MIS-C. MIS-C patients often require IMV due to hemodynamic alterations with less respiratory impairment. This is consistent with our series in which only one out of four patients diagnosed with MIS-C was categorized as having mild PARDS, while four out of five COVID-19 patients without MIS-C were stratified into moderate-severe PARDS. The Pediatric COVID-19 Spanish multicenter study, in which our patients are included, reported that 6.7% of MIS-C patients were also diagnosed with PARDS, compared with 27.4% of patients not classified as MIS-C [9].

In adults, although it was initially suggested that COVID-19-ARDS could have different characteristics than classical ARDS [39], other studies have not found these differences [14,15]. We found that the proportion of patients who progressed to ARDS was higher in the COVID-19 group, although non-COVID-19 patients had more severe PARDS. However, due to sample size constraints, we cannot draw any final conclusions.

Most patients received protective mechanical ventilation strategies with low VT, plateau and driving pressures. We found no significant group differences in relation to outcomes. However, PICU deaths were two-fold higher in the COVID-19 cohort (22% vs. 10.5%, although this difference did not reach significance, *p* = 0.57), which may be explained by the inclusion of two immunocompromised patients with severe ARDS, a comorbidity associated with greater mortality. Overall PICU mortality was 14.3%, but was higher at 25% in patients with PARDS, and was 33% in patients with severe PARDS. These results parallel those reported in other series of pediatric ARDS [35]. Although mortality from COVID-19 in children is considerably lower than in adults, fatal cases are mostly associated with serious previous comorbidities [10].

Our study has limitations that may affect comparisons and generalizability. The major limitation is that our overall patient population is small, despite the fact that the population of Spain is in excess of 47 million people. However, our data are representative of the scenario in Spain, and most likely, in western Europe, during the first couple of months of the pandemic, since 64.3% of children intubated for COVID-19 in Spain [28] during the first wave (March/April 2020) are included in our series. Second, we have restricted this analysis to intubated and mechanically ventilated children. We cannot estimate the impact of the exclusion of patients receiving non-invasive respiratory support in our study, considering the initial recommendations to limit the use of non-invasive ventilation during the first wave of the pandemic and the dramatically reduced number of admissions for other respiratory infections. Third, our analysis of respiratory failure in pediatric COVID-19 patients may be biased by the subpopulation of MIS-C patients, who were primarily intubated for hemodynamic or cardiac dysfunction, with secondary mild respiratory involvement. However, we believe that we have been able to properly differentiate these patients within the whole study population, since only one patient with MIS-C developed mild ARDS. Fourth, our study aimed to explore the respiratory mechanics of intubated patients with AHRF, but we had some missing data in 8/28 patients related to the difficulties in data collection in the midst of a major pandemic.

## 5. Conclusions

In conclusion, in this multicenter prospective study, children with AHRF due to SARS-CoV-2 infection had fewer comorbidities and better oxygenation than patients with non-COVID-19 AHRF. Unlike the adult population, progression to severe ARDS is uncommon.

## Figures and Tables

**Table 1:** Demographics and general clinical data from 28 patients with acute respiratory hypoxemic failure receiving invasive mechanical ventilation. Patients were stratified by COVID-19 infection.

	Non-COVID *n* = 19	COVID-19 *n* = 9	Total *n* = 28	*p* Value
Demographics				
Age, median (P_25_–P_75_), mo	15 (3–84)	106 (6–122)	26 (4.75–108)	0.168
Males/females (% males)	10/9 (52.6)	8/1 (88.9)	18/10 (64.3)	0.098
Weight, Kg	10 (4.05–23.5)	37(7.4–42)	11.1 (6.3–34.5)	0.061
PBW, Kg	10.4 (3.95–23)	32.3 (7.5–38.8)	13 (5.9–32.75)	0.140
Comorbidities				
Any comorbidity	15 (78.9%)	4 (44.4%)	19 (67.8%)	0.097
Chronic neurologic disease	6 (31.6%)	0	6 (21.4%)	0.136
Congenital heart disease	3 (15.8%)	0	3 (10.7%)	0.530
Cancer	3 (15.8%)	0	3 (10.7%)	0.530
Immune suppression	3 (15.8%)	2 (22.2%)	5 (17.8%)	1
Chronic lung disease	3 (15.8%)	2 (22.2%)	5 (17.8%)	1
Prematurity	4 (21%)	0	4 (14.3%)	0.273
Other	0	1 (11.1%)	1 (3.6%)	0.321
Etiological factor of AHRF				
Pneumonia or LRTI	8 (42.1%)	6 (66.7%)	14 (50%)	0.420
Sepsis/septic shock	3 (15.8%)	1 (11.1%)	4 (14.3%)	1
Non-septic shock	0	1 (11.1%)	1 (3.6%)	0.321
Neurologic dysfunction	3 (15.8%)	1 (11.1%)	4 (14.3%)	1
Surgery	2 (10.5%)	0	2 (7.1%)	1
Aspiration	2 (10.5%)	0	2 (7.1%)	1
Drowning	1 (5.3%)	0	1 (3.6%)	1
Time from PICU admission to intubation				0.769
<24 h	11(57.9%)	4 (44.4%)	15 (53.6%)	
24–48 h	5 (26.3%)	4 (44.4%)	9 (32.1%)	
>48 h	3(15.8%)	1(11.2%)	4(14.3%)	
Severity of illness				
PRISM III	7 (3–17)	7 (2–11)	7 (3–16)	0.693
PELOD 2	5 (4.5–9)	6 (6–7)	6 (4.7–7.5)	0.941
On vasoactive medication *	13 (68.4%)	7 (77.8%)	20 (71.4%)	
Inotropic Score	49 (15–57.5)	50 (30.5–83.0)	49.5 (20.2–65.6)	0.781
Prior respiratory support				
HFNC	4 (21.1%)	4 (44.4%)	8 (28.6%)	0.371
NIV	9 (47.4%)	2 (22.2%)	11 (39.3%)	0.249
Oxigenation at onset				
PaO_2_/FiO_2_ **	95 (65.5–133)	150 (105–220)	106 (66–150)	**0.046**
Oxygenation index **	15.9 (11–28.4)	9.3 (6.7–10.6)	11.2 (8.8–23.9)	**0.01**
PALICC groups, %	7/19 (36.8%)	5/9 (55.5%)	12/28 (42.8%)	**0.045**
Mild	0	1 (11.1%)	1 (3.6%)	
Moderate	0	2(22.2%)	2 (7.1%)	
Severe	7 (36.8%)	2(22.2%)	9 (32.1%)	
Length of stay, days				
PICU	16 (11–26)	19 (12–23)	16.5 (12–26)	0.902
Hospital	24 (15–49.5)	23 (22–31)	23.5 (19–41)	0.749

* Given vasoactive medication at any point within AHRF. ** PaO_2_/FIO_2_ and OI include values (6 patients without arterial line) derived from non-invasive (SpO_2_-based) analogies (SpO_2_/FIO_2_ and OSI), which have been converted to PaO_2_/FIO_2_ and OI using published equations [19]. P_25_–P_75_, 25th and 75th interquartile range; mo, months; PBW, predicted body weight. Values are expressed as median and P_25_–P_75_ unless indicated otherwise. AHRF, Acute Hypoxemic Respiratory Failure; ARDS, Acute Respiratory Distress Syndrome; HFNC, high flow nasal cannula; LRTI, lower respiratory tract infection; NIV, noninvasive ventilation; OI, oxygenation index; OSI, oxygenation/saturation index; PALICC, Pediatric Acute Lung Injury Consensus Conference; PARDS, Pediatric Acute Respiratory Distress Syndrome; PBW, predicted body weight; PELOD 2, Pediatric Logistic Organ Dysfunction 2 score; PICU, pediatric intensive care unit; PRISM III, Pediatric Risk of Mortality III score.

**Table 2:** Physiological outcomes (blood gas, hemodynamic data, and fluid balance) over time. Data collected during the first three days in the Pediatric Intensive Care Unit for the two cohorts of patients (COVID-19 and non-COVID-19).

	At Study Entry	Day 1	Day 2	Day 3
pH				
Non-COVID-19 (*n* = 14)	7.29 (7.28, 7.37)	7.34 (7.3, 7.38)	7.37 (7.34, 7.42)	7.39 (7.32, 7.44)
COVID-19 (*n* = 8)	7.24 (7.22, 7.31)	7.32 (7.26, 7.37)	7.35 (7.31, 7.4)	7.38 (7.34, 7.42)
Mean difference (*CI* 95%)	−0.04 (−0.11, 0.02)	−0.03 (−0.09, 0.04)	−0.02 (−0.09, 0.04)	−0.01 (−0.08, 0.05)
*p* value	0.18	0.44	0.54	0.72
PaCO_2_, mm Hg				
Non-COVID-19 (*n* = 13)	56 (48, 71)	53 (40, 61)	56 (43, 59)	51 (43, 57)
COVID-19 (*n* = 6)	43.5 (40, 46)	46 (39, 55)	43 (39.5, 46)	49.5 (43, 56)
Mean difference (*CI* 95%)	−12.2 (−23.7, −0.5)	−6.8 (−18.3, 4.8)	−13.2 (−24.8, 1.6)	−1.5 (−13.1, 10.1)
*p* value	**0.04**	0.25	**0.03**	0.8
Lactate (mmol/L)				
Non-COVID-19 (*n* = 17)	3.4 (1.2–2.7)	2.4 (1.3–2.6)	1.7 (1.1, 2.1)	1.6 (1–2.1)
COVID-19 (*n* = 9)	1.7 (0.8–2.1)	1.2 (1–1.3)	1.4 (1.2–1.7)	1.4 (1.2–1.7)
Mean difference (*CI* 95%)	−1.7 (−3.4, −0.1)	−1.2 (−2.9, 0.5)	−0.3 (−2–1.3)	−0.2 (−1.9, 1.4)
*p* value	**0.04**	0.16	0.71	0.78
Fluid balance (mL/Kg)				
Non-COVID-19 (*n* = 9)	12.4 (−21, 26)	27.9 (−0.2, 22.8)	9.6 (−13.5, 19.6)	−4.8 (−16.7, 4)
COVID-19 (*n* = 8)	18.6 (6.5, 44.4)	1.6 (−10.9, 27.5)	−9.8 (−20.5, 7.5)	−13.4 (−15.8, −1.8)
Mean difference (*CI* 95%)	6.2 (−25.6, 38.05)	−26.3 (−58.1, 5.5)	−19.4 (−51.2, 12.4)	−8.6 (−40.4, 23.2)
*p* value	0.7	0.11	0.23	0.6

**Table 3:** Ancillary treatments for the total cohort of 28 patients, 9 COVID and 19 non-COVID-19, during the first three days in the Pediatric Intensive Care Unit.

	Non-COVID-19 (*n* = 19)	COVID-19 (*n* = 9)	All (*n* = 28)	*p* Value
Neuromuscular blockade *				
At study entry	12/19 (67.9%)	4/9 (44.4%)	16/28 (57.1%)	0.432
Day 1	10/18 (55.6%)	8/9 (88.9%)	18/27 (66.7%)	0.193
Day 2	10/18 (55.6%)	7/9 (77.8%)	17/27 (63%)	0.406
Day 3	8/17 (47.1%)	5/9 (55.6%)	13/26 (50%)	1
Prone positioning	3/19 (15.8%)	3/9 (33.3%)	6/28 (21.4%)	0.352
Inhaled nitric oxide	6/19 (31.6%)	1/9 (11.1%)	7/28 (25%)	0.371
ECMO	2/19 (10.5%)	1/9 (11.1%)	3/28 (10.7%)	1
Corticosteroids	11/19 (57.9%)	8/9 (88.9%)	19/28 (67.9%)	0.195

* Neuromuscular blockade (NMB) is detailed according to onset and within the first three days of AHRF. ECMO: extracorporeal membrane oxygenation.

**Table 4:** Outcome measures. Categorical variables are expressed as numbers (%), and continuous variables are expressed as median (P_25_–P_75_ interquartile range). PICU, Pediatric intensive care unit.

	Non-COVID-19 (*n* = 19)	COVID-19 (*n* = 9)	All (*n* = 28)	*p* Value
Pneumothorax after initiating mechanical ventilation	2 (10.5%)	1 (11.1%)	3 (10.7%)	1
PICU mortality	2 (10.5%)	2 (22.2%)	4 (14.3%)	0.574
Hospital mortality	3 (15.8%)	2 (22.2%)	5 (17.9%)	1
Ventilator-free days	(18–24)	21 (5.5–23)	19 (5.5–24)	0.749
PICU length of stay, days	16 (11–26)	19 (12–23)	16.5 (12–26)	0.902
Hospital length of stay, days	24 (15–49.5)	23 (22–31)	23.5 (9–41)	0.749

## Data Availability

Not applicable.

## Supplementary Materials

The following are available online at https://www.mdpi.com/article/10.3390/jcm10194301/s1, Electronic Supplementary Material (ESM) 1, ESM: “Strengthening the Reporting of Observational Studies in Epidemiology (STROBE)” guidelines for observational cohort studies. ESM 2: details of PANDORA-CHILD. Table S1: Ventilatory settings for the total cohort. Table S2: Ancillary treatments for the total cohort.

## Appendix A. Members of the PANDORA-CHILD Network

Yolanda M López-Fernández, Javier Pilar Orive. Hospital Universitario de Cruces, Bizkaia.

Amelia Martínez-de-Azagra Garde, Angeles García-Teresa. Hospital Niño Jesús. Madrid.

Eider Oñate-Vergara, Nerea Ovelar Zubiaga. Complejo Hospitalario Donosti. Gipuzkoa

Ana Gómez-Zamora. Hospital Universitario La Paz. Madrid.

Laura Herrera-Castillo, Laura Butragueño. Hospital Universitario Gregorio Marañón. Madrid.

Juan I. Sánchez, Ana Llorente de la Fuente. Hospital Universitario 12 de Octubre. Madrid.

Susana Beatriz Reyes, María Miñambres-Rodriguez. Hospital Universitario Virgen de la Arrixaca. Murcia.

Mikel Mendizabal, María Amores Torres. Complejo Hospitalario de Navarra.

Francisco Fernández Carrión, Sira Fernández de Miguel. Hospital Universitario de Salamanca.

Antonio Rodríguez-Núñez, Javier Trastoy-Quintela, Susana Rey-García. Hospital Universitario de Santiago.

Julio Parrilla-Parrilla, Manuel Fernández-Elías. Hospital Universitario Virgen del Rocío. Sevilla.

David Arjona, María José Pérez Durán. Hospital Universitario Virgen de la Salud. Toledo

Marta Brezmes, Lorena Bermúdez. Hospital Clínico Universitario de Valladolid.

Patricia Rodriguez-Campoy, Lorena Estepa-Pedregosa. Hospital Universitario Puerta del Mar, Cádiz.

Luis Pérez-Baena, José Sebastián León-González. Hospital Universitario de Tenerife.

María García-González, Isabel del Blanco. Hospital Universitario de Burgos.

Paula Madurga-Revilla, Juan Pablo Iñiguez. Hospital Universitario Miguel Servet. Zaragoza.

Rocío Núñez-Borrero, Sira Alonso-Graña, Félix Castillo-Ferrer. Hospital Universitario Las Palmas de Gran Canaria.

José Manuel González-Gómez, Antonio Morales, Alexandra Fernández-Yuste. Hospital Regional Universitario de Málaga.

Alberto Medina, Juan Mayordomo-Colunga. Hospital Central de Asturias.

Ana Coca Pérez, Cesar Pérez-Caballero Macarrón. Hospital Universitario Ramón y Cajal. Madrid.

Ignacio Ibarra de la Rosa. Hospital Universitario Reina Sofía. Córdoba.

Jesús Villar, Jesús M. González-Martín. Research Unit, Hospital Universitario Dr. Negrín Las Palmas de Gran Canaria.
