# Supplementary material for: Acute Hypoxemic Respiratory Failure in Children at the Start of COVID-19 Outbreak: A Nationwide Experience
Source: J Clin Med. 2021 Sep 22;10(19):4301. doi: 10.3390/jcm10194301 (PMC8509571; doi:10.3390/jcm10194301)
Supplement: Supplementary file 1 [file jcm-10-04301-s001.zip › PANCOVID-Online SI.pdf]

ONLINE SUPPORTING INFORMATION

Acute Hypoxemic Respiratory Failure in Children at the start of COVID-19 outbreak: A nationwide experience

[1] Yolanda M. López-Fernández, MD
[2] Amelia Martínez-de-Azagra, MD
[3] José M. González-Gómez, MD
[4] Cesar Perez-Caballero Macarrón, MD, PhD
[5] María García-Gonzalez, MD
[6] Julio Parrilla-Parrilla, MD
[7] María Miñambres-Rodriguez, MD
[8] Paula Madurga-Revilla, MD
[9] Ana Gómez-Zamora, MD
[10] Patricia Rodríguez-Campoy, MD
[11,12] Juan Mayordomo-Colunga, MD
[13] Laura Butragueño-Laiseca, MD
[14] Rocío Núñez-Borrero, MD
[15] Jesús M. González-Martín, PhD
[16,17] Arthur S. Slutsky, MD
[12,17,18] Jesús Villar, MD, PhD
*For the Prevalence and Outcome of Acute Hypoxemic Respiratory Failure in Children (PANDORA-CHILD) Network**

From
(1) Pediatric Intensive Care Unit, Department of Pediatrics, Cruces University Hospital, Biocruces-Bizkaia Health Research Institute, Barakaldo, Bizkaia, Spain;
(2) Pediatric Intensive Care Unit, Niño Jesús University Hospital, Madrid, Spain;
(3) Pediatric Intensive Care Unit, Hospital Regional Universitario de Málaga, Spain;
(4) Pediatric Intensive Care Unit, Hospital Universitario Ramón y Cajal, Madrid, Spain;
(5) Pediatric Intensive Care Unit, Complejo Hospitalario de Burgos, Burgos, Spain;
(6) Pediatric Intensive Care Unit, Hospital Universitario Virgen del Rocío, Sevilla, Spain;
(7) Pediatric Intensive Care Unit, Hospital Universitario Virgen de la Arrixaca, Murcia, Spain;
(8) Pediatric Intensive Care Unit, Hospital Universitario Miguel Servet, Zaragoza, Spain;
(9) Pediatric Intensive Care Unit, Hospital Universitario La Paz, Madrid, Spain;
(10) Pediatric Intensive Care Unit, Hospital Universitario Puerta del Mar, Cádiz, Spain;
(11) Pediatric Intensive Care Unit, Hospital Universitario Central de Asturias, , Instituto de Investigación del Principado de Asturias, Oviedo, Spain;
(12) CIBER de Enfermedades Respiratorias, Instituto de Salud Carlos III, Madrid, Spain;
(13) Pediatric Intensive Care Unit, Hospital Universitario Gregorio Marañón, Madrid, Spain;
(14) Pediatric Intensive Care Unit, Hospital Universitario Materno-infantil, Las Palmas de Gran Canaria, Spain;
(15) Research Unit, Hospital Universitario Dr. Negrín, Las Palmas de Gran Canaria, Spain;
(16) Interdepartmental Division of Critical Care Medicine, University of Toronto, Toronto, Canada;
(17) Keenan Research Center for Biomedical Sciences at the Li Ka Shing Knowledge Institute, St. Michael's Hospital, Toronto, Canada;
(18) Multidisciplinary Organ Dysfunction Evaluation Research Network (MODERN), Research Unit, Hospital Universitario Dr. Negrín, Las Palmas de Gran Canaria, Spain.
(*) Members of the PANDORA-CHILD Network are listed in the Appendix of Main Manuscript.

ELECTRONIC SUPPLEMENTARY MATERIAL 1

STROBE Statement—Checklist of items that should be included in reports of ***cohort studies***

	Item No	Recommendation
**Title and abstract**	1	(*a*) Indicate the study's design with a commonly used term in the title or the abstract. **Pages 1 and 3**
		(*b*) Provide in the abstract an informative and balanced summary of what was done and what was found. **Page 3**
**Introduction**		
Background/rationale	2	Explain the scientific background and rationale for the investigation being reported. **Page 4**
Objectives	3	State specific objectives, including any prespecified hypotheses **Pages 4 and 5**
**Methods**		
Study design	4	Present key elements of study design early in the paper **Page 5**
Setting	5	Describe the setting, locations, and relevant dates, including periods of recruitment, exposure, follow-up, and data collection. **Pages 5-7 and Electronic Supplementary Material 2**
Participants	6	(*a*) Give the eligibility criteria, and the sources and methods of selection of participants. Describe methods of follow-up. **Pages 5-7**
		(*b*) For matched studies, give matching criteria and number of exposed and unexposed **Page 5 and 6**
Variables	7	Clearly define all outcomes, exposures, predictors, potential confounders, and effect modifiers. Give diagnostic criteria, if applicable **Page 6 and 7**
Data sources/ measurement	8*	For each variable of interest, give sources of data and details of methods of assessment (measurement). Describe comparability of assessment methods if there is more than one group **Pages 6 and 7**
Bias	9	Describe any efforts to address potential sources of bias **Page 8**
Study size	10	Explain how the study size was arrived at **Page 8**
Quantitative variables	11	Explain how quantitative variables were handled in the analyses. If applicable, describe which groupings were chosen and why **Page 7**
Statistical methods	12	(*a*) Describe all statistical methods, including those used to control for confounding **Page 8**
		(*b*) Describe any methods used to examine subgroups and interactions **Page 8**
		(*c*) Explain how missing data were addressed **Page 8**
		(*d*) If applicable, explain how loss to follow-up was addressed **Not applicable**
		(*e*) Describe any sensitivity analyses **Not applicable**
**Results**		
Participants	13*	(a) Report numbers of individuals at each stage of study—eg numbers potentially eligible, examined for eligibility, confirmed

		eligible, included in the study, completing follow-up, and analysed **Pages 8 and 9**
		(b) Give reasons for non-participation at each stage **Pages 8 and 9**
		(c) Consider use of a flow diagram **Page 9**
Descriptive data	14*	(a) Give characteristics of study participants (eg demographic, clinical, social) and information on exposures and potential confounders **Pages 9 and 10, Table 1**
		(b) Indicate number of participants with missing data for each variable of interest **Pages 9 and 10, Table 2 and Table S1**
		(c) Summarise follow-up time (eg, average and total amount) **Page 15**
Outcome data	15*	Report numbers of outcome events or summary measures over time **Page 15**
Main results	16	(*a*) Give unadjusted estimates and, if applicable, confounder-adjusted estimates and their precision (eg, 95% confidence interval). Make clear which confounders were adjusted for and why they were included **Pages 10-14**
		(*b*) Report category boundaries when continuous variables were categorized **Pages 10-14**
		(*c*) If relevant, consider translating estimates of relative risk into absolute risk for a meaningful time period **Not applicable**
Other analyses	17	Report other analyses done—eg analyses of subgroups and interactions, and sensitivity analyses **Not applicable**
**Discussion**		
Key results	18	Summarise key results with reference to study objectives **Page 17**
Limitations	19	Discuss limitations of the study, taking into account sources of potential bias or imprecision. Discuss both direction and magnitude of any potential bias **Pages 20 and 21**
Interpretation	20	Give a cautious overall interpretation of results considering objectives, limitations, multiplicity of analyses, results from similar studies, and other relevant evidence **Pages 17-20**
Generalisability	21	Discuss the generalisability (external validity) of the study results **Pages 17-20**
**Other information**		
Funding	22	Give the source of funding and the role of the funders for the present study and, if applicable, for the original study on which the present article is based **Not applicable**

*Give information separately for exposed and unexposed groups.

ELECTRONIC SUPPLEMENTARY MATERIAL 2. METHODS

Study design and participants

Study period: this study includes a specific analysis of children admitted with acute hypoxemic respiratory failure (AHRF) and invasive respiratory support in 16 Spanish PICUs during the first wave (March/April 2020) of SARS-CoV-2 pandemic. The parent study (**P**revalence **and** **O**utcomes of Acute Hypoxemic **R**espiratory F**a**ilure in **child**ren, PANDORA-CHILD) has been enrolling patients from October 2019, and will continue until September 2021. The present sub-analysis is restricted to March-April 2020. Patients have been enrolled during 6 periods of two consecutive months (1 October to 30 November 2019, 1 February to 31 March 2020, 1 June to 31 July 2020, 1 December 2020 to 31 January 2021, 1 April to 31 May 2021 and 1 August to 31 September 2021). After the declaration of the COVID-19 pandemic in March 2020, it was decided to extend the study by one month and to perform a sub-analysis within March/April 2020 to investigate the physiologic characteristics and ventilatory management of AHRF in intubated children (including COVID-19 patients) from participating centers and following the same criteria and methodology as for the PANDORA-child study. The institutional ethics committee of the coordinating centers were informed and approved the amendment to extend the study to April 2020.

Informed consent: this study was considered an audit, and informed consent was waived, but three sites required written parental consent as per the institution´s policy: Niño Jesús Children´s Hospital, Madrid; La Paz University Hospital, Madrid; and Reina Sofía University Hospital, Córdoba.

Geographical catchment areas: 22 PICUs in Spain agreed to participate. However, during the study period, 6 of those PICUs served as adult ICUs due to the COVID-19 pandemic. The remaining 16 ICUs showed a marked decrease in pediatric admissions, but still continued to screen and recruit patients for the parent study. Therefore, eligible patients came from 8

geographical catchment areas of Spain. The total population of the area covered by participating hospitals was 21.465.569, and the total number of residents <16 years of age was 3.383.356 [1].

Data Collection

All data were collected using standardized forms. Demographics, comorbidities, reason for initiation of IMV, arterial blood gases, laboratory, radiographic, hemodynamic and ventilator data were collected at study entry and during the first three days of AHRF diagnosis (T0 or time of inclusion into the study, 24 hours, days 2 and 3). In cases where invasive arterial sampling was not available, the pulse oximetric saturation (SpO_2/FiO_2 ratio) was calculated as a surrogate of PaO_2/FiO_2 ratio (18). A SpO_2/FiO_2 ratio of 264 corresponded with a PaO_2/FiO_2 ratio of 300 [2]. Chest images (chest radiographs, lung ultrasound or computed tomography) were assessed as diagnostic test at onset of AHRF as well as evaluated daily for the presence or absence of infiltrates, atelectasis, acute pulmonary edema, pleural effusion or pneumothorax. Additional data included vasopressor requirement, fluid balance and ancillary therapies. Standard definitions for pediatric sepsis and organ failure were used [3].

General Management

Some of the patients received corticosteroids, nitric oxide, prone positioning, high frequency oscillatory ventilation (HFOV) or extracorporeal membrane oxygenation (ECMO) as adjunctive therapies. Fluid resuscitation and vasopressor administration were individualized. It was recommended to maintain hemoglobin between 7 and 10 g/dL[4].

SUPPLEMENTARY REFERENCES

1. Spanish National Statistics Institute: 2020 census data Ine.int 2020 [accessed 2021 Jan 31] https://www.ine.es/jaxiT3/Tabla.htm?t=31304.
2. Khemani RG et al; Pediatric Acute Lung Injury and Sepsis Network Investigators (PALISI). Comparison of SpO2 to PaO2 based markers of lung disease severity for children with acute lung injury. Crit Care Med, 2012, **40**, 1309-16.
3. Goldstein B, Giroir B, Randolph A; and the Members of the International Consensus Conference on Pediatric Sepsis. International pediatric sepsis consensus conference: Definitions for sepsis and organ dysfunction in pediatrics. Pediatr Crit Care Med, 2005, **6**, 2-8.
4. Valentine SL et al. Consensus recommendations for RBC transfusion practice in critically ill children from the Pediatric Critical Care Transfusion and Anemia Expertise Initiative. Pediatr Crit Care Med, 2018, **19**, 884-898.
