# Supplementary material for: Acute Hypoxemic Respiratory Failure in Children at the Start of COVID-19 Outbreak: A Nationwide Experience
Source: J Clin Med. 2021 Sep 22;10(19):4301. doi: 10.3390/jcm10194301 (PMC8509571; doi:10.3390/jcm10194301)
Supplement: Supplementary file 1 [file jcm-10-04301-s001.zip › Supplemental table 1.pdf]

Supplemental Table S1. Ventilatory settings for the total cohort of 28 patients, 9 COVID and 19 non-COVID-19 patients during the first three days in the Pediatric Intensive Care Unit.

	At study entry	***Day 1***	***Day 2***	***Day 3***
**VT, mL/kg PBW**				
Non-COVID-19(N=19)	7.1 (6-9.2)	7.3 (6-9)	7.9 (5.7-10)	6.9 (4.6-9.7)
COVID-19 (N=9)	7.5 (7.1-8.2)	7.8 (6.7-8.9)	7.5 (7-8.3)	7.9 (4.4-9)
All (N=28)	7.4 (6.1-9)	7.6 (6.1-9)	7.6 (5.9-9.5)	7.7 (5-8.9)
P value	0.962	0.803	0.662	0.878
**PEEP, cm H_20**				
Non-COVID-19(N=19)	8 (5.5-9.5)	10 (7-12)	8 (6-10)	7 (6.9-8)
COVID-19 (N=9)	7 (6-8)	9 (6-10)	9 (7-10)	9 (6-10)
All (N=28)	8 (6-9)	10 (6.5-11.5)	8 (6.5-10)	7 (6.5-9)
P value	0.566	0.567	0.533	0.666
**Plateau pressure, cmH_20**				
Non-COVID-19(N=13)	27 (21.2-29.7)	25 (21-30)	23 (20-29)	21 (20-25)
COVID-19 (N=7)	23 (21-25.7)	23 (21-28.5)	21 (20-23)	22.5 (20-25)
All (N=20)	25.5 (21-29)	24.5 (21-30)	23 (20-25)	21 (20-25)
P value	0.362	0.660	0.303	1
**Driving pressure, cmH_20**				
Non-COVID-19(N=13)	16 (11.2-21.5)	14 (13-19)	15 (13-19)	13 (12-14)
COVID-19 (N=7)	16 (13.2-18)	13 (13-18.5)	13.5 (13-15)	13 (13-14.5)
All (N=20)	16 (13-20.5)	13.5 (13-19)	14 (13-17)	13 (12.5-14.5)
P value	1	0.935	0.304	1

VT, Tidal volume; PBW, predicted body weight, PEEP, Positive end-expiratory pressure.
