# Supplementary material for: Acute Hypoxemic Respiratory Failure in Children at the Start of COVID-19 Outbreak: A Nationwide Experience
Source: J Clin Med. 2021 Sep 22;10(19):4301. doi: 10.3390/jcm10194301 (PMC8509571; doi:10.3390/jcm10194301)
Supplement: Supplementary file 1 [file jcm-10-04301-s001.zip › Supplemental table 2 .pdf]

Supplemental Table S2. Ventilatory settings over the first three days after admission in the Pediatric Intensive Care Unit for the two cohorts of patients (COVID-19 and non-COVID-19).

	At study entry	*Day 1*	*Day 2*	*Day 3*
**VT, mL/kg PBW**				
Non-COVID-19(N=15)	7.8 (6.1, 9.2)	7.4 (6.1, 8.9)	7.4 (5.6, 9.8)	7 (4.5, 9.8)
COVID-19 (N=7)	7.5 (7.2, 8.1)	7.6 (6.2, 8.5)	7.8 (7.3, 8.5)	7.8 (7.5, 8.1)
Mean difference (CI 95%)	-0.25 (-2.07, 1.56)	0.25 (-1.56, 2.07)	0.46 (-1.35, 2.27)	0.79 (-1.03, 2.6)
P value	0.79	0.78	0.62	0.4
**PEEP, cm $H_2$0**				
Non-COVID-19(N=16)	9 (6, 9)	10 (7, 12)	8 (7, 10)	8 (7, 8)
COVID-19 (N=9)	7.5 (6, 8)	9 (6, 10)	9 (7, 10)	9 (6, 10)
Mean difference (CI 95%)	-1.07 (-3.43, 1.29)	-0.86 (-3.22, 1.5)	0.69 (-1.67, 3.05)	0.57 (-1.79, 2.94)
P value	0.38	0.48	0.57	0.64
**Plateau pressure, cm$H_2$0**				
Non-COVID-19(N=8)	26 (23.5, 29)	26 (21, 30)	25 (21, 29)	23 (20, 26)
COVID-19 (N=4)	22.5 (21, 23)	23 (2.5, 23)	21.5 (20, 23)	21 (20, 21)
Mean difference (CI 95%)	-3.38 (-9.19, 2.44)	-3.13 (-8.94, 2.69)	-3.38 (-9.19, 2.44)	-2.38 (-8.19, 3.44)
P value	0.26	0.29	0.26	0.42
**Driving pressure, cm$H_2$0**				
Non-COVID-19(N=8)	17 (11, 20)	16 (13, 19)	16 (13, 19)	14 (13, 15)
COVID-19 (N=4)	15.5 (13, 16)	14.5 (13, 15)	13 (13, 13.5)	12.5 (12, 14)
Mean difference (CI 95%)	-1.25 (-6.01, 3.51)	-1.5 (-6.26, 3.26)	-2.75 (-7.5, 2.01)	-1.63 (-6.39, 3.14)
P value	0.61	0.54	0.26	0.5
**Crs, mL/Kg/cm$H_2$0**				
Non-COVID-19(N=8)	0.55 (0.36, 0.62)	0.47 (0.42, 0.54)	0.45 (0.39, 0.5)	0.52 (0.46, 0.64)
COVID-19 (N=4)	0.54 (0.51, 0.58)	0.55 (0.46-0.65)	0.66 (0.61, 0.7)	0.69 (0.56-0.72)
Mean difference (CI 95%)	-0.01(-0.22, 0.19)	0.09 (-0.12, 0.3)	0.21 (-0.04, 0.37)	0.09 (-0.12, 0.3)
P value	0.91	0.4	0.05	0.12

VT, Tidal volume; PBW, predicted body weight, PEEP, Positive end-expiratory pressure. Crs, compliance of the respiratory system
