# Supplementary material for: Acute Hypoxemic Respiratory Failure in Children at the Start of COVID-19 Outbreak: A Nationwide Experience
Source: J Clin Med. 2021 Sep 22;10(19):4301. doi: 10.3390/jcm10194301 (PMC8509571; doi:10.3390/jcm10194301)
Supplement: Supplementary file 1 [file jcm-10-04301-s001.zip › Supplemental table 3.pdf]

Supplemental Table S3. Blood gas and hemodynamic data, fluid balance and need of renal replacement therapy or transfusion for the total cohort of 28 patients, 9 COVID and 19 non-COVID-19 patients during the first three days in the Pediatric Intensive Care Unit.

	At study entry	*Day 1*	*Day 2*	*Day 3*
**pH**				
Non-COVID-19 (N=18)	7.30 (7.19-7.34)	7.35(7.31-7.39)	7.39 (7.34-7.42)	7.39 (7.31-7.43)
COVID-19 (N=9)	7.28 (7.25-7.31)	7.34 (7.27-7.39)	7.35 (7.31-7.41)	7.38 (7.34-7.42)
All (N=27)	7.3 (7.22-7.37)	7.34 (7.3-7.39)	7.38 (7.34-7.41)	7.39 (7.32-7.43)
**P value**	0.519	0.666	0.698	1
**$PaCO_2$, mm Hg**				
Non-COVID-19 (N=15)	48 (46.5-63)	49.5 (40.7-59.7)	54 (43-58.7)	47 (43-57)
COVID-19 (N=7)	41 (40 - 45)	43 (39.5-53.5)	41 (38.5-45.5)	46 (43-54.5)
All (N=22)	47.5 (40-51.5)	48 (40-57)	45 (42-55)	46.5 (43-57.5)
**P value**	0.071	0.411	**0.03**	0.968
**Lactate (mmol/L)**				
Non-COVID-19 (N=18)	2 (1.1-3.2)	1.6 (1.1-2.6)	1.5 (1-2.1)	1.5 (1-2.1)
COVID-19 (N=9)	1.4 (0.8-2.1)	1.2 (1-1.3)	1.3 (1.2-1.4	1.4 (1.2-1.7)
All (N=27)	1.8 (0.9-2.5)	1.4 (1-2.1)	1.3 (1.1-1.9)	1.4 (1-1.7)
**P value**	0.228	0.094	0.588	0.626
**Fluid balance (ml/Kg)**				
Non-COVID-19 (N=11)	12 (-16, 40)	4.2 (-8.8, 19.7)	6.7 (-9.4, 30.8)	0.2 (-18.3, 23.4)
COVID-19 (N=8)	11.7 (6.7, 44.5)	-1.8 (-10.9, 27.5)	-11 (-32.1, 1.7)	-9.3 (-24.5, -2.1)
All (N=19)	12 (-7, 46)	0.65 (-9, 22.2)	1.7 (-16.7, 25.1)	-5 (-23, 11.3)
**P value**	0.519	0.666	0.698	1
**RRT**				
Non-COVID-19	2/19 (10.5%)	3/18 (16.7%)	3/18 (16.7%)	3/17 (17.6%)
COVID-19	0/9	0/9	0/9	0/9
All	2/28 (7.1%)	3/27 (11.1%)	3/27 (11.1%)	3/26 (11.5%)
**P value**	1	0.529	0.529	0.529
**Blood transfusion**				
Non-COVID-19	10/19 (52.6%)	5/18 (27.8%)	2/18 (11.1%)	3/16 (18.8%)
COVID-19	2/9 (22.2%)	1/9 (11.1%)	0/9	0/9
All	12/28 (42.9%)	6/27 (22.2%)	2/27 (7.4%)	3/25 (12%)
**P value**	0.223	0.628	0.538	0.280

RRT, renal replacement therapy.
